# Present status and upcoming prospects of hedgehog pathway inhibitors in small cell lung cancer therapy

**DOI:** 10.1186/1750-9378-8-17

**Published:** 2013-05-22

**Authors:** Syed Hassan Abbas Naqvi, Syed Hassan Shiraz Naqvi, Muhammad Yasin Bandukda, Syed Mumtaz Ali Naqvi

**Affiliations:** 1 Dow Medical College, Dow University of Health Sciences, Baba-e-Urdu Road, Karachi, Pakistan

**Keywords:** Hedgehog, Inhibitors, Lung, Cancer

## Abstract

Lung cancer is an important etiology of malignant mortality worldwide with global statistics indicating over 1 million deaths annually. Although there have been advances in cytotoxic chemotherapy, the prognosis after treatment still remains poor. Remarkably, recent studies on the molecular level are creating the possibility to hamper lung cancer by inhibiting the hedgehog pathway. Currently, hedgehog pathway inhibitors include IWP-2, cyclopamine and aprotinin. However, Vismodegib is a new upcoming prospect which has shown positive results while undergoing clinical trials. If approved, it may lead to a novel class of anti-cancer therapy for patients seeking treatment for small cell lung cancer.

## Letter to the editor

Lung cancer is an important etiology of malignant mortality worldwide with global statistics indicating over 1 million deaths each year [1]. Despite advances in cytotoxic chemotherapy in addition to traditional treatments, the prognosis still remains poor due to limited efficacy. Remarkably, recent studies on the molecular level have created the possibility to detect, prevent and treat small cell lung cancer in its initial stage by inhibiting a novel pathway involved in embryogenesis known as the Hedgehog (Hh) pathway.

The Hh pathway involves a family of secreted proteins that participate in the regulation of cell growth, differentiation and survival [2,3]. Furthermore, these unique proteins have also been implicated in promoting stem cell proliferation in adults. However, if they are abnormally hyperactivated in adult tissues through sporadic mutations or other mechanisms, they may play a crucial role in tumorigenesis of various organs especially the lungs [4-6]. Amongst these, the ones recently discovered to contribute in small cell lung cancer [7,8] development are Smoothened (SMO), Rab23, platelet-derived growth factor alpha (PDGFRα), hedgehog interacting protein (HIP) and hepatocyte nuclear factor 3-beta (HNF3β) [5]. For this reason, the use of Hh pathway inhibitors in lung cancer have strongly been considered.

Hh pathway inhibitors are a novel class of therapeutic agents that specifically target proteins which are involved in the regulation of the Hh pathway. Currently, the inhibitors include IWP-2, cyclopamine and aprotinin; each one altering one of the mutated afore mentioned proteins to prevent malignancy by inhibiting wnt/β-catenin pathway [9], blocking hedgehog signaling by binding to heptahelical bundle of smoothened (smo) [10] and as protease inhibitor [11], respectively. In addition, itraconazole (Sporanox) are a different class of drugs which have also shown potential; having the same action as cyclopamine except through a different mechanism [12] while arsenic trioxide antagonizes the Hedgehog pathway by preventing ciliary accumulation thus reducing stability of the Gli2 transcriptional effector [13]. Although these drugs have been successful in treating invasive cancers, their role in lung cancer therapy remains to be determined. Many studies have discussed the potential role of HHIs in small cell lung cancer but a lot of these drugs are still in their trial phase. Various studies discussed new strategies of lung cancer management with a possible role of HHI in future [14,15].

Interestingly, Vismodegib (trade name Erivedge) is a new upcoming prospect which has shown positive results for inhibiting the Hh pathway in different cancer while undergoing clinical trials in the United States [16]. It acts to suppress Hh signaling by binding to and interfering with smoothened, a membrane protein that provides positive signals to the Hh signaling pathway [17]. Moreover, this drug is extensively bound to plasma proteins which accounts for its longer half-life [18]. Its recent approval for the treatment of advanced basal cell carcinoma (BCC) has now made it a viable candidate in lung cancer therapy. However, like other drugs Vismodegib also has side effects including muscle spasms, weight loss, dysguesia, alopecia and constipation [19]. Nevertheless, if Vismodegib is approved by the Food and Drug Administration (FDA) for small cell lung cancer, it may lead to a novel class of anti-cancer therapy for patients seeking treatment for lung cancer. Moreover, these advancements may potentially lead to a cascade of new clinical trials with more Hh inhibiting drugs being tested for their safety and efficacy. Optimistically, these alone, or in combination with traditional therapy, may provide the breakthrough oncologists need in their struggle to find a definite cure. However, for this notion to be successful, collaborative multiinstitutional efforts will be necessary.

## Abbreviations

Hh: Hedgehog; SMO: Smoothened; PDGFRα: Platelet-derived growth factor alpha; HIP: Hedgehog interacting protein; HNF3β: Hepatocyte nuclear factor 3-beta; BCC: Basal cell carcinoma; FDA: Food and drug administration.

## Competing interests

The authors declare that no conflict of interest exists.

## Authors’ contributions

AN was involved in choosing the topic and drafting the original manuscript. SN, MB and MN were involved in critically reviewing the manuscript. All authors have read and approved the manuscript. The authors did not receive any financial support/grants.
